# Clinical case analysis and finite element analysis of Beavis type II avulsion fractures of the calcaneal tuberosity

**DOI:** 10.3389/fsurg.2026.1765823

**Published:** 2026-09-22

**Authors:** Changpeng Cao, Faxin Cao, Xiyao Wang, Haiyang Xing, Gang Wang

**Affiliations:** 1 Department of Orthopedics, China-Japan Union Hospital of Jilin University, Changchun, China; 2 First Affiliated Hospital of Jilin University, Changchun, China; 3 Department of Orthopedics, Second Affiliated Hospital of Jilin University, Changchun, China

**Keywords:** Beavis II avulsion fracture of calcaneal tuberosity, calcaneal tuberosity avulsion fractures, finite element analysis, fixation methods, osteoporosis

## Abstract

**Background:**

Calcaneal tuberosity avulsion fractures are primarily caused by intense traction of the Achilles tendon. Clinical treatment remains challenging, with a high failure rate. Current research mainly focuses on injury mechanisms, retrospective analyses, and case discussions, while biomechanical experimental evidence is still lacking. Therefore, selecting the most appropriate internal fixation method to achieve satisfactory outcomes remains a key issue to be addressed.

**Methods:**

This study employed finite element analysis to compare the fixation efficacy of five internal fixation techniques for type II calcaneal tuberosity fractures. Clinical cases involving treatment failure were retrospectively reviewed to explore the management process and underlying causes, aiming to provide a mechanical basis and reference for future clinical practice.

**Results:**

The 180° annular plate was identified as the optimal fixation method for treating such fractures. In addition, the Achilles tendon tension band technique significantly reduced the risk of treatment failure.

**Conclusion:**

For type II calcaneal tuberosity avulsion fractures, the 180° annular plate provides the best biomechanical performance, while the Achilles tendon tension band technique also effectively lowers the failure rate. These findings offer valuable mechanical evidence to guide clinical decision-making and improve surgical outcomes.

## 1 Introduction

Calcaneal fractures are the most common tarsal fractures, accounting for 60% of tarsal fractures and 1.5% of all fractures (1, 2). Calcaneal tuberosity avulsion fractures are rare, accounting for approximately 1.3%–2.7% of all calcaneal fractures (3, 4). Lee et al. divided calcaneal tuberosity avulsion fractures into four types (5). Type Ⅰ is a simple extra-articular avulsion fracture and is the most common calcaneal tuberosity avulsion fracture. Type Ⅱ is a “beak” type fracture, and the fracture line is obliquely downward from the subtalar joint toward the Achilles tendon insertion. Type Ⅲ is an avulsion fracture caused by the superficial fibers of the middle third of the Achilles tendon. Type Ⅳ is an avulsion fracture of the deep fibers of the Achilles tendon (3, 5). Previous studies have shown that calcaneal tuberosity avulsion fractures are closely associated with osteoporosis, diabetes, peripheral vascular disease, hypothyroidism, and other comorbidities (6, 7). Calcaneal bone content decreases with age, as do bone trabeculae, and bone loss is more obvious in women than in men (5, 8–10). A type Ⅰ fracture usually occurs due to slight trauma (such as tripping). Type III and IV fractures occur primarily as a result of intense muscle contraction with the heel fixed to the ground (5). Type Ⅱ fractures often occur when strong muscle contraction is combined with direct violence, and are most commonly seen in elderly patients with osteoporosis. Conservative treatment of calcaneal tuberosity avulsion fractures is only suitable for minor displaced fractures without damage to soft tissue, and surgical treatment is necessary for patients with soft tissue compression or large fracture displacement (11, 12).

In recent years, with the continuous advancement of digital medicine, computer-based simulation technologies have been increasingly applied to fracture injury modeling. To achieve better fracture reduction and prevent complications, fixation devices and surgical techniques have undergone continuous evolution. For calcaneal tuberosity avulsion fractures, fixation options have evolved from the initial Kirschner-wire tension bands and cannulated screws to anchor screws, microplates, external fixators, and other devices tailored to different fracture types. This study utilized finite element analysis to evaluate the biomechanical performance of various internal fixation constructs for this fracture type and conducted an in-depth analysis based on clinical failure cases. The aim was to provide a scientific basis and reference for optimizing clinical treatment protocols and formulating postoperative management strategies. By combining retrospective clinical analysis with finite element modeling, this study seeks to furnish mechanical evidence to inform future clinical decision-making.

## 2 Finite element analysis

### 2.1 Modeling

One healthy male volunteer, aged 27 years, 172 cm tall, and weighing 70 kg, with no history of lower extremity trauma or other conditions affecting bone metabolism, was selected. Informed consent was obtained, and the study was approved by the ethics committee of our hospital. The patient's whole foot was scanned using a CT scanner, and the images were saved in Digital Imaging and Communications in Medicine (DICOM) format. The data were imported into Mimics 19.0 to generate three-dimensional (3D) images. The 3D images were exported in STL format and imported into Geomagic Studio 2013, where they were segmented into meshes. Based on anatomical characteristics, the cortical and cancellous bone models of the calcaneus were reconstructed and subsequently assembled using SolidWorks 21.0. The surgical approach was determined according to the size of the fracture fragment. In accordance with the Beavis classification and the dimensional criteria described by Michael Doany for Beavis type II fractures, the fracture fragment was modeled as a beak-type fragment with a length greater than 3 cm and a height greater than 1.5 cm (Figure 1).

**Figure 1:**
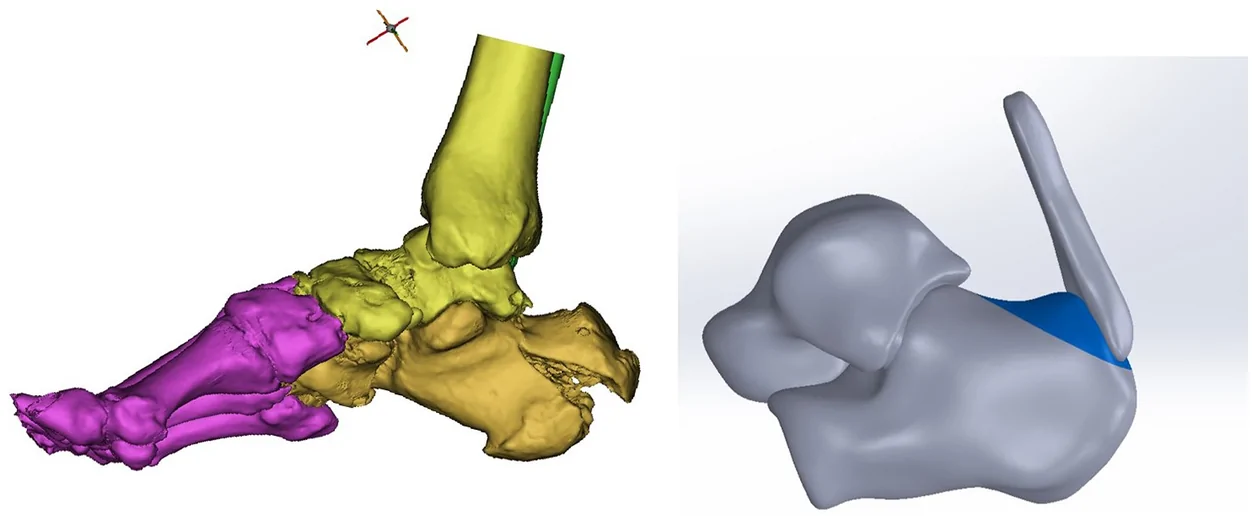
Fracture model. Left: 3D CT reconstruction of a calcaneal tuberosity avulsion fracture. Right: corresponding 3D model of the fracture.

### 2.2 Grouping

Based on existing reports and clinical treatment methods, we designed the following five fixation methods (10, 12–17): (a) 2 mm × 5.5 mm fully threaded cannulated screws (2 mm × 5.5 mm FTCS), (b) 2 mm × 5.5 mm double-head compression screws (2 mm × 5.5 mm DHCS), (c) 2 mm × 5.5 mm fully threaded cannulated screws + wire tension band (14) (2 mm × 5.5 mm FTCS + TB), (d) 3 mm × 4.5 mm fully threaded cannulated screws (3 mm × 4.5 mm FTCS), and (e) a 180° annular plate (180° AP) (12) (Figure 2).

**Figure 2:**
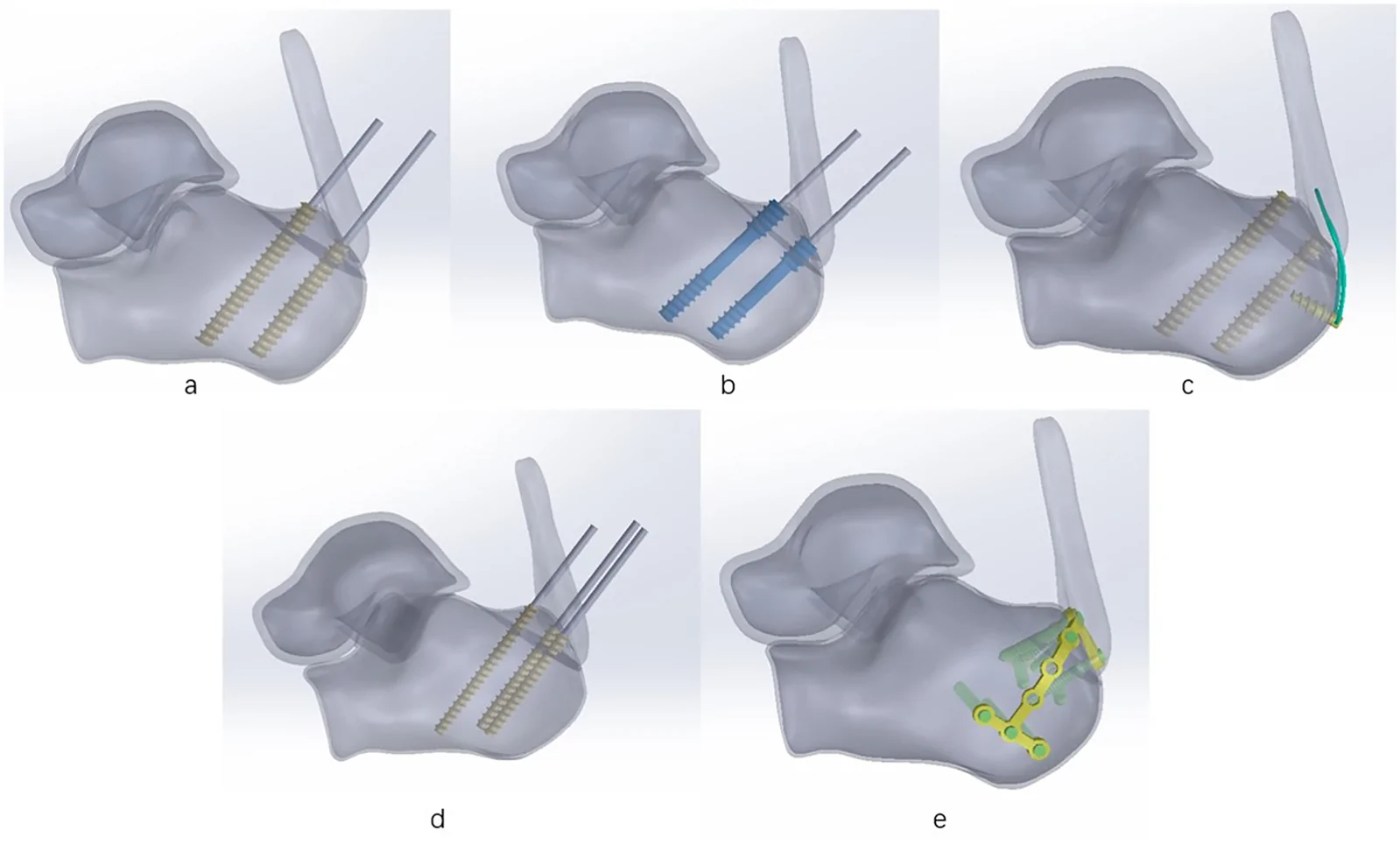
Fracture fixation models. **(a)** 2 mm × 5.5 mm FTCS; **(b)** 2 mm × 5.5 mm DHCS; **(c)** 2 mm × 5.5 mm FTCS + TB; **(d)** 3 mm × 4.5 mm FTCS; **(e)** 180° AP.

In this study, all materials, including bone and metals, were simplified as isotropic, homogeneous, and linear elastic materials. Based on literature reports, the material properties of osteoporotic cortical and cancellous bone were adopted, with specific properties detailed in Table 1 (18, 19). Appropriate mesh refinement helps improve the accuracy of experimental results and reduce computational cost. Ultimately, we determined the optimal mesh size to be 1 mm (20).

**Table 1:** Material properties used in the simulations in this study.

Material	Elastic modulus (MPa)	Poisson's ratio
Titanium alloy	1,05,000	0.31
Cortical bone (osteoporosis)	16,800	0.3
Cancellous bone (osteoporosis)	580	0.2

### 2.3 Loading and restraint

To reduce the traction effect of the Achilles tendon on the fracture fragment postoperatively, the affected limb is often immobilized in a knee-flexed and plantarflexed position, and weight-bearing is prohibited. Studies have found that when the ankle is in 20° plantarflexion, a neutral position, and 10° dorsiflexion, the tension of the Achilles tendon is 10, 30, and 120 N, respectively. Therefore, this study analyzed the following three loading conditions representing postoperative bed rest: (1) 20° plantarflexion at 10 N; (2) neutral position at 30 N; and (3) 10° dorsiflexion at 120 N (21–26). The proximal talus, calcaneocuboid joint, and plantar region were fixed, and a tensile load was applied along the direction of Achilles tendon contraction (Figure 3). The contact relationships between various materials were defined according to previous relevant studies. The cortical–cancellous bone interface was set as bonded. The contact between fracture ends was set as frictional, with a friction coefficient of 0.46. The contact between screws and bone was also set as frictional, with a coefficient of 0.3 (27, 28).

**Figure 3:**
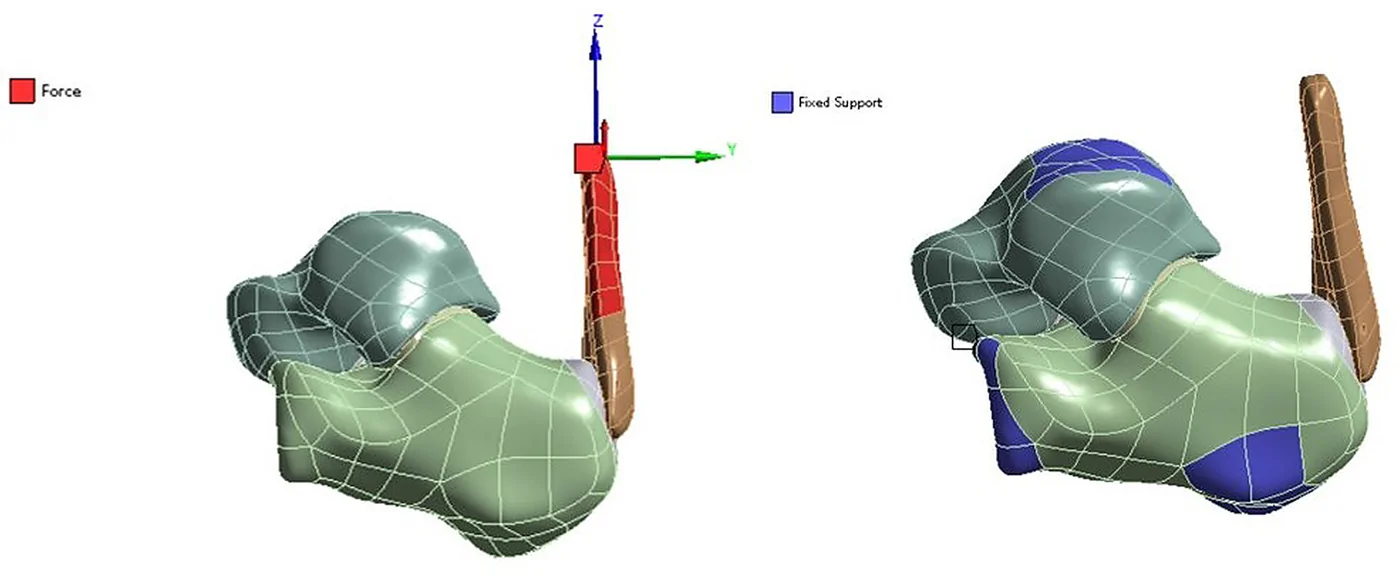
Loading and restraint. Left: loading position and direction. Right: constraint location.

### 2.4 Model validation

A load in the vertical direction was applied to the intact calcaneal model of this study, which was compared with the stiffness of the vertical structure of the calcaneus in published studies (29, 30). The validation results of this experiment were largely consistent with those of previous studies.

## 3 Results

### 3.1 Model validation results

Model verification and model validation results are shown in Figures 4 and 5, respectively.

**Figure 4:**
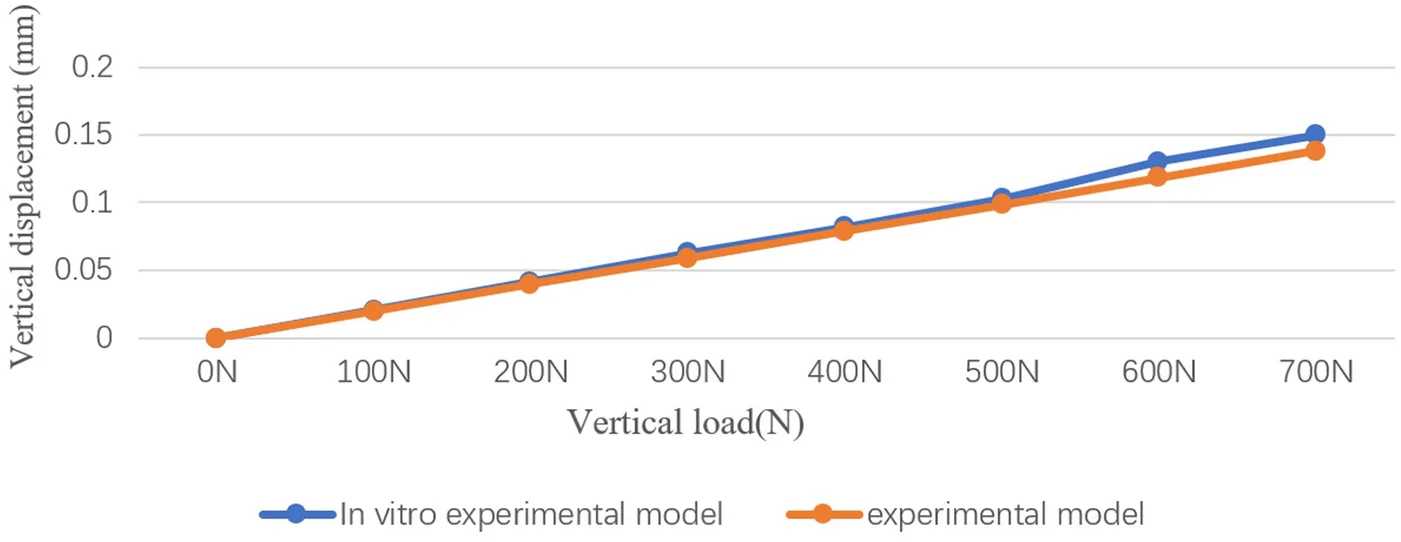
Model verification. The model shows similar mechanical behavior to previous studies.

**Figure 5:**
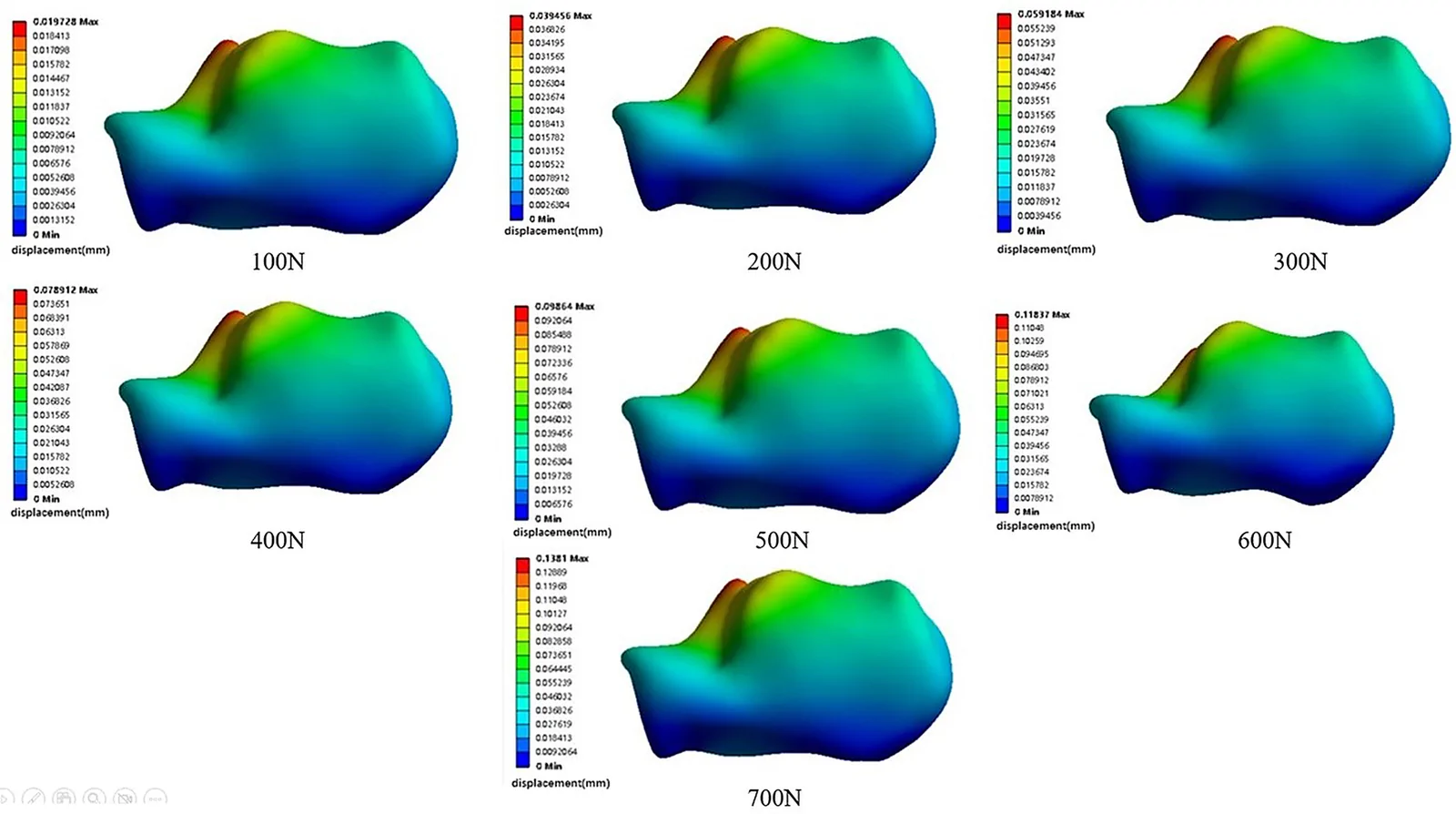
Model validation results. Von Mises stress cloud diagram for model validation (100–700 N).

### 3.2 Maximum von Mises stress and displacement cloud diagram of fracture internal fixation model

Among the five fixation constructs, group C exhibited the lowest peak von Mises stress in the implant, with a value of 49.002 MPa under the maximum load. In group E (180° annular plate), the peak stress under the maximum load was 53.97 MPa (Figures 4, 5).

Regarding bone stress, the 180° annular plate demonstrated the best stress dispersion effect on the fracture fragment, effectively avoiding local stress concentration in both the bone and implant, thereby minimizing the risk of fixation failure. Under maximum load, the von Mises stress in the bone for group E was 15.225 MPa. For groups A and C, both fixed with 5.5 mm fully threaded screws, the addition of a tension band in group C significantly reduced the traction load of the Achilles tendon on the fracture fragment, and the peak bone stress was markedly lower than that in the other three screw-only groups. In groups A, B, and E, the von Mises stresses in the calcanei and implants did not exceed the yield strength of the implant or bone.

Regarding fracture-site displacement, group E showed the smallest displacement under maximum traction load (0.059 mm). The primary reason is that the 180° annular plate converts point fixation into planar fixation, thereby enhancing fixation stability while achieving favorable stress distribution. Group C followed with 0.0834 mm, as the Achilles tendon tension band effectively reduced the tensile force on the fracture fragment, decreasing both displacement and stress. Table 2 and Figures 6–8 present the results for all five fixation models under the three loading conditions.

**Figure 6:**
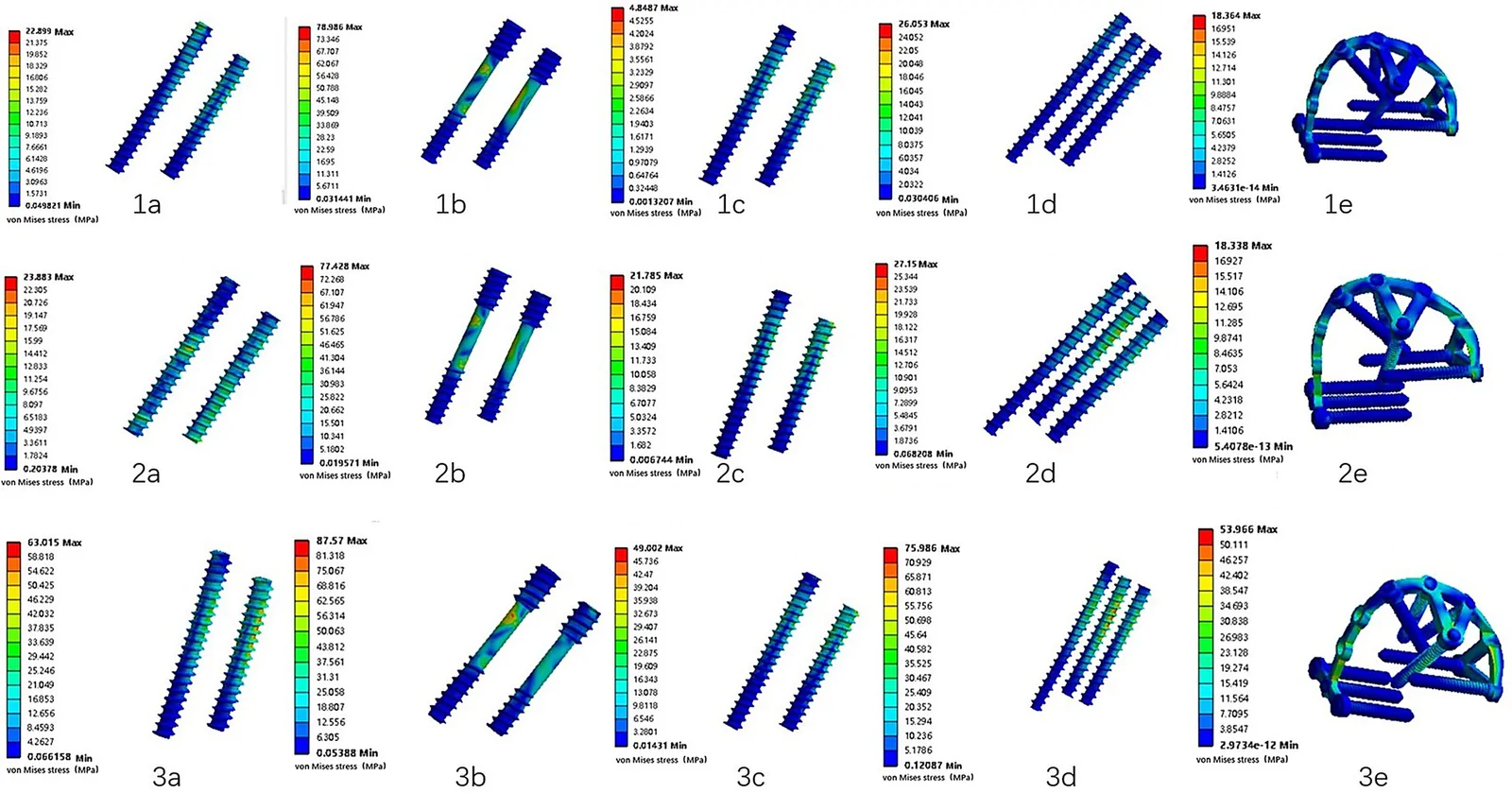
Von Mises stress of implants under loads of 10, 30, and 120 N. Panels **(a–e)** correspond to fixation models A–E as defined in Table 2.

**Figure 7:**
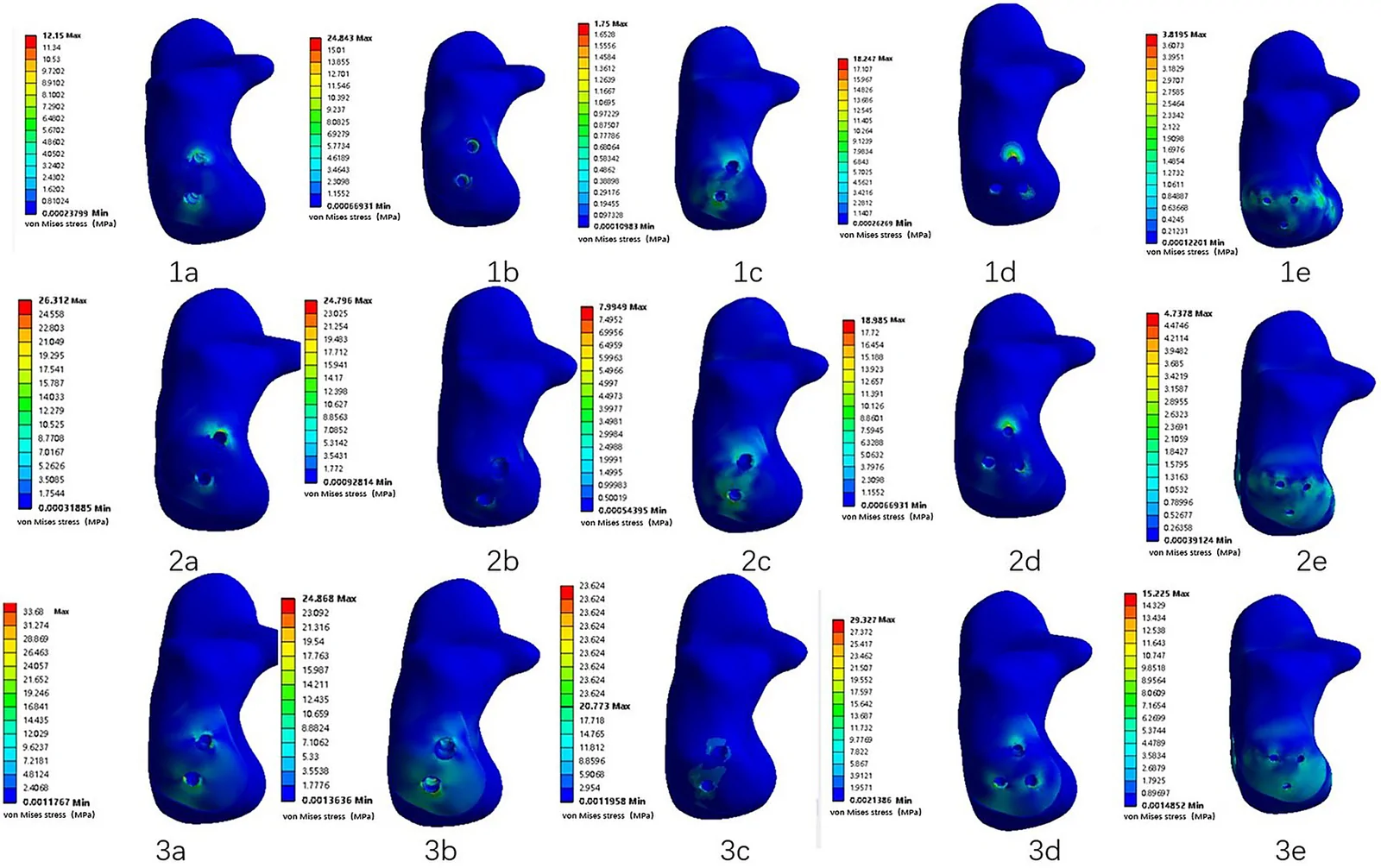
Von Mises stress of the calcaneus under loads of 10, 30, and 120 N. Panels **(a–e)** correspond to fixation models A–E as defined in Table 2.

**Figure 8:**
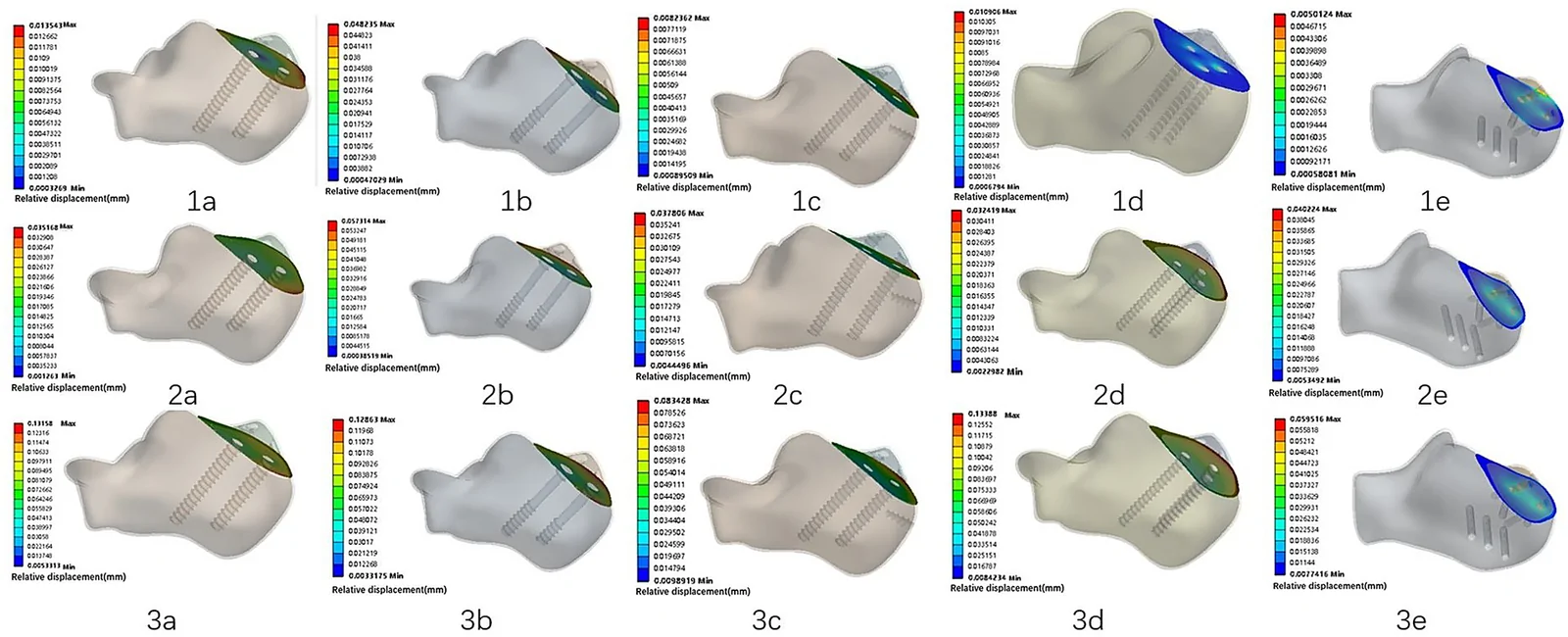
Displacement at the fracture site under loads of 10, 30, and 120 N. Panels **(a–e)** correspond to fixation models A–E as defined in Table 2.

**Table 2:** Peak von Mises stress and displacement across all fracture fixation models.

Load (N)	Parameter	A	B	C	D	E
10	Implant stress (MPa)	22.899	78.986	4.849	26.053	18.364
30	Implant stress (MPa)	23.883	77.428	21.785	27.15	18.338
120	Implant stress (MPa)	63.015	87.570	49.002	75.986	53.966
10	Calcaneus stress (MPa)	12.150	24.843	1.750	18.247	3.820
30	Calcaneus stress (MPa)	26.312	24.796	7.995	18.985	4.738
120	Calcaneus stress (MPa)	33.68	24.868	23.624	29.327	15.225
10	Displacement (mm)	0.014	0.048	0.008	0.011	0.005
30	Displacement (mm)	0.035	0.057	0.038	0.032	0.040
120	Displacement (mm)	0.132	0.129	0.083	0.134	0.060

A = 2 mm × 5.5 mm FTCS; B = 2 mm × 5.5 mm DHCS; C = 2 mm × 5.5 mm FTCS + TB; D = 3 mm × 4.5 mm FTCS; E = 180° AP.

### 3.3 Peak von Mises stress of the implants

Figure 6 presents the peak von Mises stresses of the five fixation models under loads of 10, 30, and 120 N. At all load levels, model C exhibited the lowest stress values—only 4.85 MPa at 10 N, rising to 49.00 MPa at 120 N. Model B consistently showed the highest stresses, ranging from 78.99 MPa at 10 N to 87.57 MPa at 120 N. Models A, D, and E demonstrated intermediate stress levels: at 120 N, model A reached 63.02 MPa, model D 75.99 MPa, and model E 53.97 MPa. As the load increased from 10 to 120 N, implant stress rose in all models. Model C showed the largest relative increase (approximately 10-fold), yet its absolute values remained far lower than those of the other models. In contrast, model B exhibited the smallest increase (approximately 11%), but its stresses stayed persistently high.

### 3.4 Peak von Mises stress of the calcaneus

Peak von Mises stress results for the calcaneus are shown in Figure 7. Under a 10 N load, models C (1.75 MPa) and E (3.82 MPa) showed the lowest calcaneal stress. At 30 N, models C (7.99 MPa) and E (4.74 MPa) remained the lowest. At 120 N, model E exhibited the lowest calcaneal stress (15.23 MPa), followed by model C (23.62 MPa), while model A had the highest (33.68 MPa). Notably, model B displayed a unique stress-stable behavior, with its calcaneal stress varying minimally with increasing load (approximately 24.8 MPa). Model D showed a calcaneal stress of 29.33 MPa at 120 N, which was higher than that of models C and E. Overall, models C and E provided better stress protection to the calcaneus than the other models.

### 3.5 Fracture site displacement

The results for fracture site displacement are presented in Figure 8. Under all load conditions, model E (180° annular plate) consistently exhibited the smallest displacement values: 0.005 mm at 10 N and only 0.0595 mm at 120 N. Model C showed the second smallest displacement, with 0.0834 mm at 120 N. Models A, B, and D demonstrated similarly high displacements at 120 N, reaching 0.1316, 0.1286, and 0.1339 mm, respectively. As the load increased, displacement increased in all models; model E showed a relatively modest increase (from 0.005 mm to 0.0595 mm), whereas models A, B, and D exhibited more pronounced increases. These findings indicate that models E and C are significantly superior to the screw-only constructs in controlling relative displacement at the fracture site and maintaining fixation stability.

## 4 Clinical case report

This study collected 21 patients with calcaneal tuberosity avulsion fractures in a large general hospital from March 2017 to December 2022. Data on the patients' age, gender, fracture block size, surgical method, and fracture treatment outcome were collected and are presented in Table 3. Written informed consent for the publication of this study was obtained from all patients. All methods in this study were in accordance with the rights of the patient and were authorized by the patient. Ethical approval was provided by the Ethics Committee of China-Japan Union Hospital of Jilin University.

**Table 3:** Clinical data of the cases.

No.	Sex	Age	L/R	Internal fixation devices	Outcome
1	Female	67	L	4.5 mm (F)	Fail
2	Female	54	L	5.5 mm (H)	Healing
3	Male	31	R	5.5 mm (H)	Healing
4	Female	80	R	4.5 mm (H)	Fail
5	Male	41	R	4.5 mm (F)	Healing
6	Female	65	R	3.5 mm (F)	Fail
7	Male	51	R	4.5 mm (F)	Healing
8	Female	53	L	5.5 mm (F)	Healing
9	Male	40	L	4.5 mm Herbert	Healing
10	Female	78	L	5.5 mm (F)	Fail
11	Male	22	R	5.5 mm (F)	Healing
12	male	50	L	4.5 mm (F)	Healing
13	Female	82	L	5.5 mm Herbert	Healing
14	Male	36	R	4.5 mm Herbert	Healing
15	Male	50	L	4.5 mm (F)	Healing
16	Female	40	L	5.5 mm (F)	Healing
17	Female	81	L	4.5 mm Herbert	Healing
18	Male	31	R	5.5 mm Herbert	Healing
19	Male	23	L	Tension band +4.5 mm (F)	Healing
20	Male	60	L	4.5 mm (F)	Healing
21	Male	65	L	4.5 mm (F)	Healing

F, fully threaded cannulated screws; H, partially threaded screw; L/R, left/right. Total of 21 cases, including four failures (failure rate 19.05%).

### 4.1 Case 1

A 67-year-old female patient sustained a left calcaneal tuberosity avulsion fracture without significant trauma. The fracture fragment was displaced on the third day after surgery (Figure 9).

**Figure 9:**
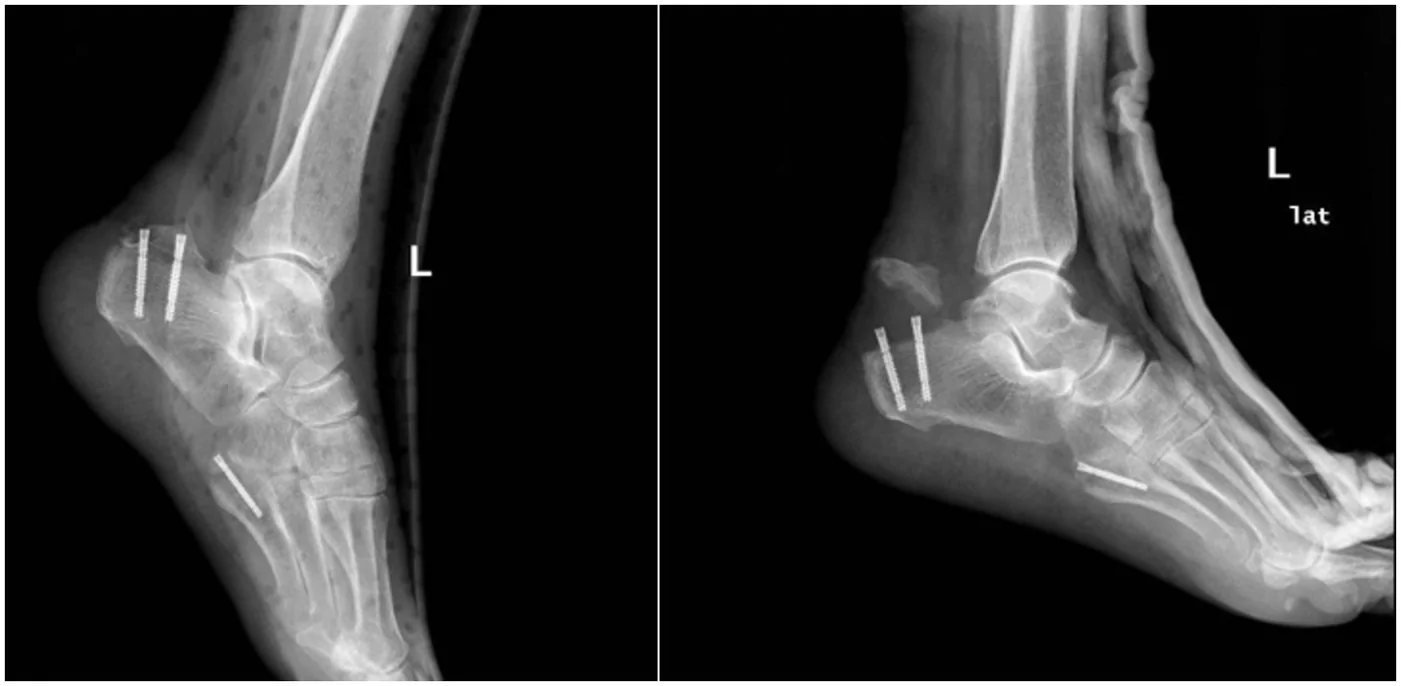
Case 1. Left: postoperative image. Right: failed treatment image.

### 4.2 Case 2

An 80-year-old female patient with a right calcaneal tuberosity avulsion fracture was fixed with a 4.5-mm semi-threaded screw. After the operation, the affected limb was fixed in flexion and plantarflexion positions with a cast, but the fracture fragment was partially displaced because the patient fell to the ground 5 weeks after the operation (Figure 10).

**Figure 10:**
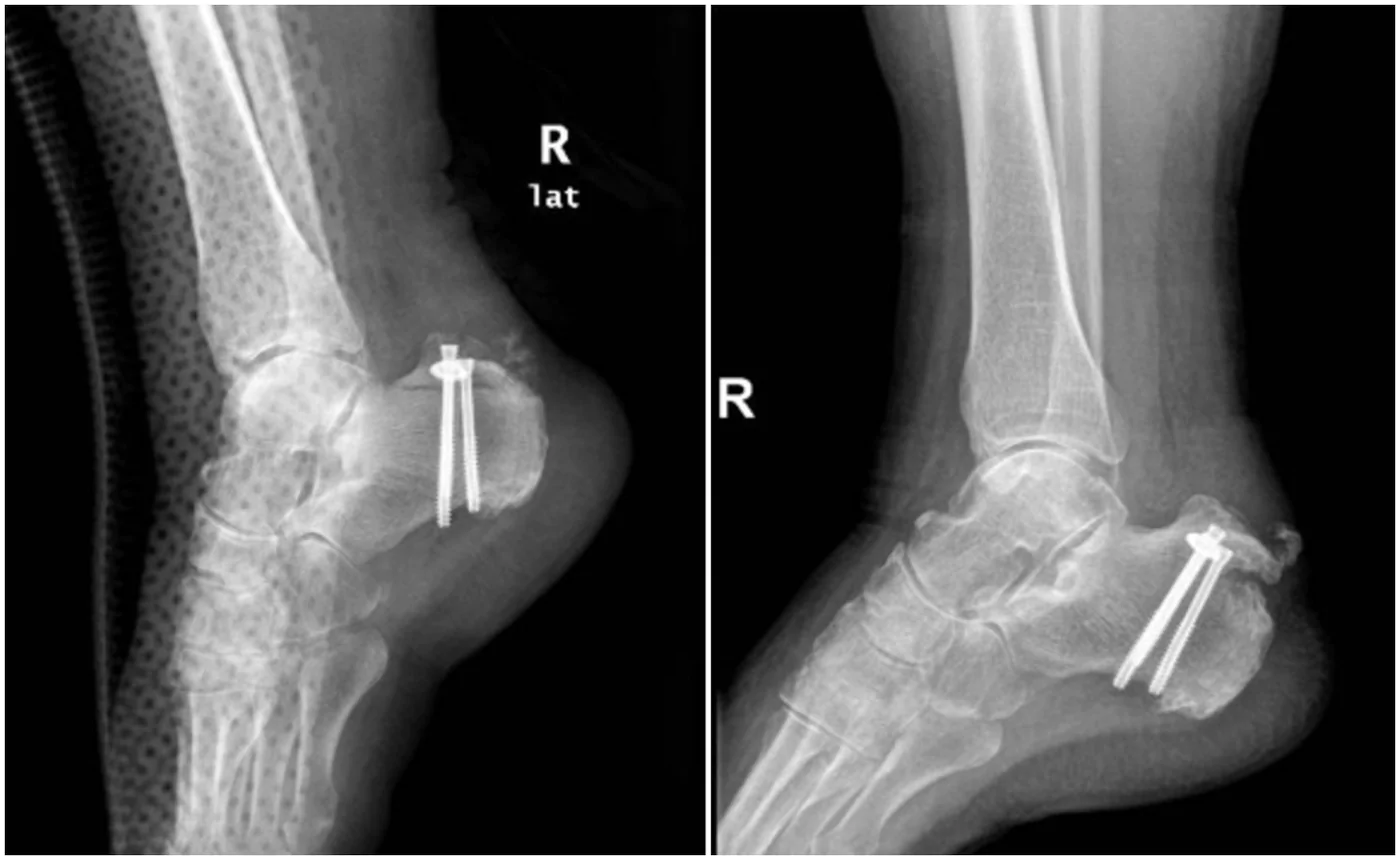
Case 2. Left: postoperative image. Right: failed treatment image.

### 4.3 Case 3

A 65-year-old female patient with a right calcaneal tuberosity avulsion fracture was fixed with a 3.5-mm semi-threaded screw. After the operation, the affected limb was fixed in the flexion and plantar flexion positions with a cast. However, the patient removed the cast by herself 3 days after the operation, resulting in obvious displacement of the fracture block and failure of the internal fixation device (Figure 11).

**Figure 11:**
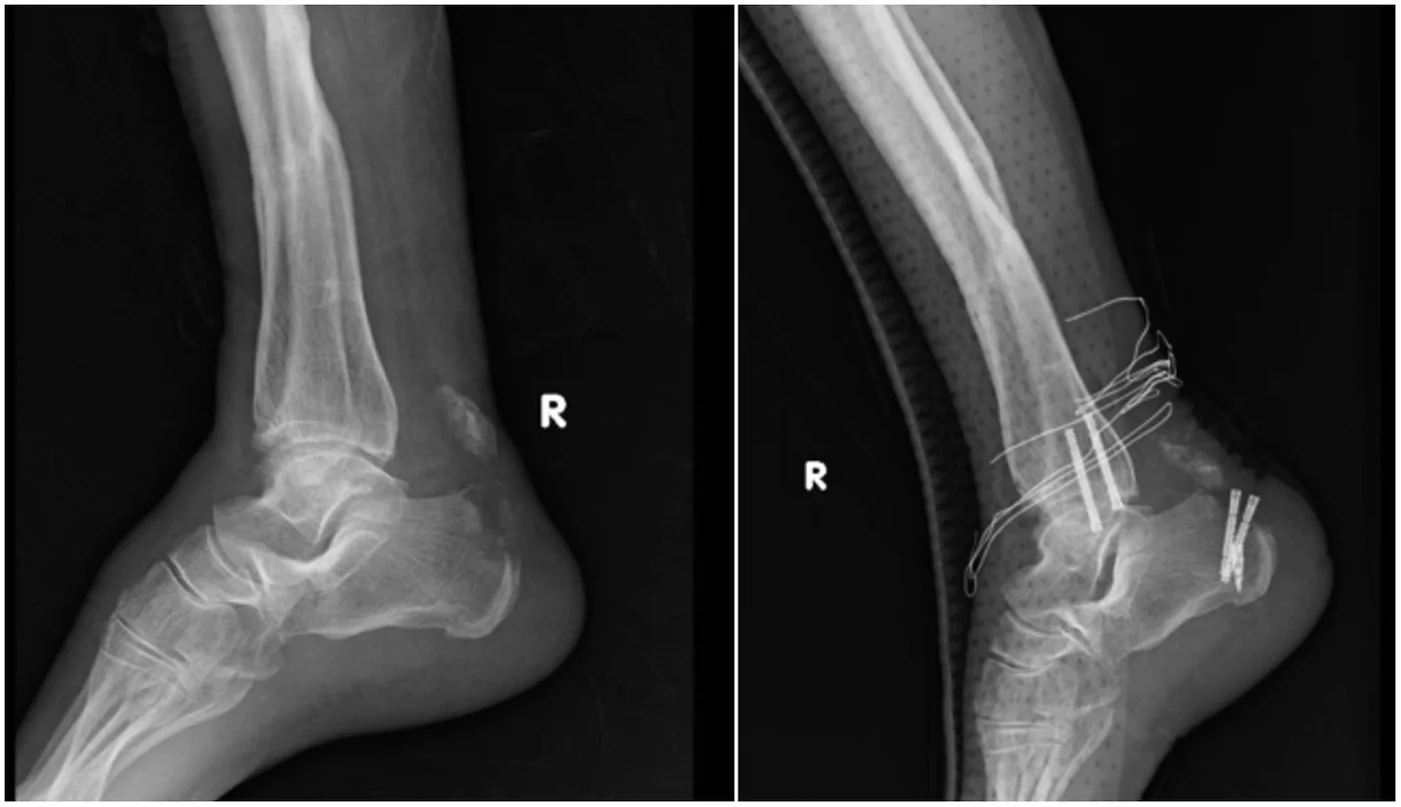
Case 3. Left: preoperative image. Right: treatment failure image.

### 4.4 Case 4

A 78-year-old female patient with a left calcaneal tuberosity avulsion fracture was fixed with 3.5- and 4.5-mm full-thread screws, and the affected limb was fixed in flexion and plantar flexion positions without plaster after the operation. At 2 weeks after the operation, the fracture fragment was displaced and the internal fixation device failed (Figure 12).

**Figure 12:**
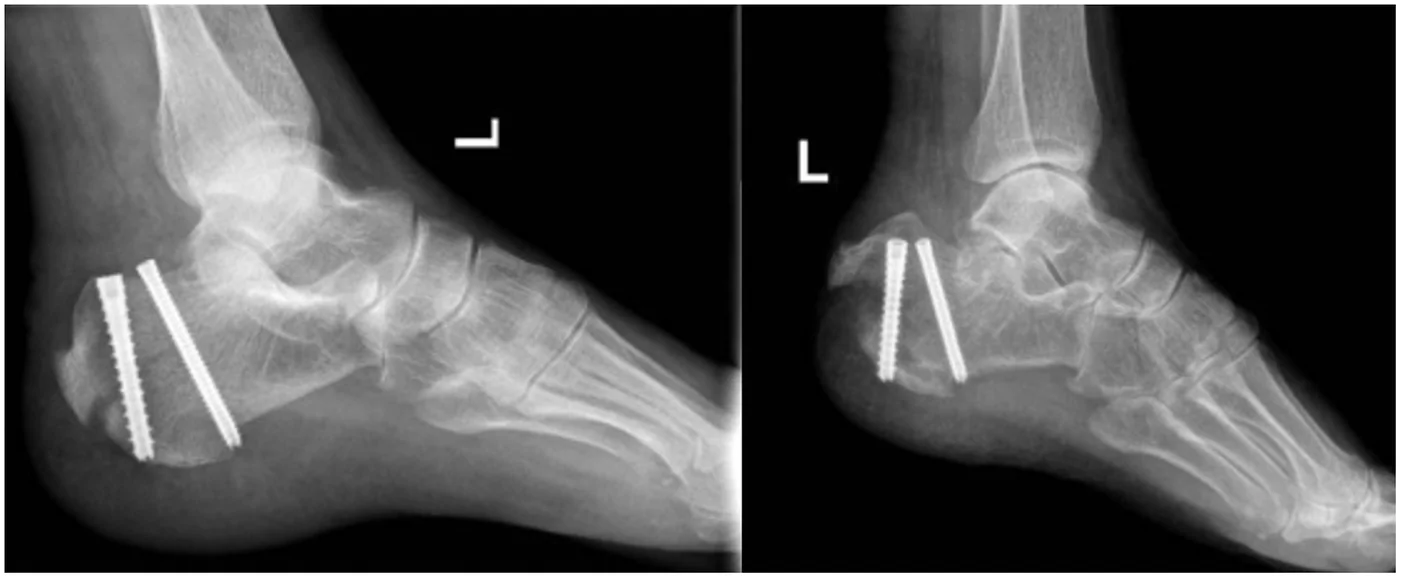
Case 4. Left: postoperative image. Right: failed treatment image.

Inappropriate surgical technique (Case 1) and premature weight-bearing or poor patient compliance (Cases 2–4) can easily lead to internal fixation failure. Both standardized surgical treatment and diligent postoperative management are critically important for successful outcomes.

## 5 Discussion

A calcaneal tuberosity avulsion fracture is a very difficult type of calcaneal fracture to treat. The postoperative complications of calcaneal tuberosity avulsion fractures mainly include internal fixation failure, heel pain, and postoperative wound infection. The Achilles tendon is one of the most powerful tendons in the human body; during normal walking, the maximum load on the Achilles tendon is three times one’s body weight, and during strenuous activity, it can reach up to 5,000 N (31, 32). The strong traction of the Achilles tendon and the stress concentration caused by osteoporosis and the failure of internal fixation are the challenges in the treatment of such fractures. Studies have shown that the incidence of infection and skin necrosis after a calcaneal fracture is as high as 30% (33–35). This is particularly true for Beavis type II calcaneal fractures; owing to the limited soft tissue envelope around the calcaneus, severe swelling, and poor terminal blood supply, the fracture fragment is displaced proximally by Achilles tendon traction, compressing the posterior heel skin and leading to local soft tissue ischemia and necrosis (34, 36–38).

Currently, a variety of fixation methods are available for large fracture fragments. Two 5.5-mm fully threaded compression cannulated screws, double-headed compression Herbert screws, or three 4.5-mm fully threaded compression cannulated screws placed perpendicular to the fracture line are commonly used (36, 38, 39). Since failure of this fracture type is mostly attributable to the traction effect of the Achilles tendon on the fracture fragment, some scholars have suggested using an Achilles tendon tension band in conjunction with screw fixation to reduce this traction effect (14). However, the majority of existing literature on this fracture type consists of retrospective clinical analyses, and biomechanical evidence is lacking. Consequently, there is no unified standard regarding which internal fixation method achieves a satisfactory fixation effect.

Previous studies have demonstrated that when the von Mises stress of the bone surrounding an implant exceeds 56 MPa, loosening and eventual failure of the internal fixation are highly likely to occur (40). According to existing research, orthopedic titanium alloy implants undergo irreversible changes when von Mises stress exceeds 450 MPa, leading to fixation failure (40). The data from this study show that group C had the lowest peak von Mises stress in the implant (49.002 MPa), while group E was 53.966 MPa. Yao et al. (20) published a study in *Scientific Reports* comparing the mechanical properties of multiple internal fixation schemes for Sanders type II calcaneal body fractures. They found that under a 1,400 N axial load, the maximum von Mises stress of the lag screw was 491.0 MPa, the frame locking plate was 772.7 MPa, and the T-shaped locking plate was as high as 931.8 MPa. Although the loading conditions differ, the above data support the advantage of multi-screw systems in reducing implant stress—they effectively disperse the load through multi-point, multi-dimensional fixation, avoiding stress concentration on a single component. Notably, Ni et al. (41) found that although locking plates can provide maximum stiffness, they also produce the highest peak von Mises stress. This finding is consistent with our observation that implant stress in group E (annular plate) was slightly higher than that in group C, suggesting that while plate fixation offers good stability, it often underperforms optimized screw configurations in controlling stress concentration. Furthermore, this study found that group E (180° annular plate) had the lowest bone von Mises stress (15.225 MPa). Our analysis attributes this to the circular plate transforming point fixation into planar fixation, thereby effectively avoiding local stress concentration. This value is considerably lower than the bone stress range (2.6–502.6 MPa) reported in the systematic review by Zdero et al. (42), highlighting the unique advantage of circular plates in stress distribution.

From a mechanical mechanism perspective, traditional lateral calcaneal plates often cause stress concentration on one side due to incomplete conformity with the irregular anatomical shape of the calcaneus. In contrast, the 180° annular plate achieves more uniform load transfer by increasing the contact area between the fixation device and the bone surface. Han et al. (43) compared the biomechanical performance of the following four models: percutaneous sustentaculum screw fixation, intramedullary nailing, a locking compression plate, and a minimally invasive plate. They found that the sustentaculum screw model had the lowest peak stress in the calcaneus. This study similarly verifies the effectiveness of optimized fixation systems in improving bone stress distribution.

In terms of maximum displacement at the fracture site, group E performed best (0.059 mm), followed by group C (0.0834 mm). We believe that the advantage of group E stems from the 180° annular plate transforming point fixation into planar fixation, thereby enhancing resistance to displacement. Han et al.'s study also found that the maximum displacement of fracture fragments in all internal fixation models was below the reference threshold of 150 μm, which is consistent with the finding in this study that the data from both groups were far less than 1 mm. The systematic review by Zdero et al. (42) summarized the fracture site displacement range reported in the literature as 0–11.24 mm. The displacement values in this study fall within the lowest range, further validating the excellent performance of groups E and C in controlling micromotion.

Our results clearly demonstrate that, of groups A and C, both fixed with 5.5-mm fully threaded screws, group C, which incorporated a tension band, had significantly lower peak bone stress compared with the other screw-only groups. This finding has important clinical implications: the Achilles tendon inserts into the calcaneal tuberosity and constitutes a key deforming force and source of traction after fracture. Nagura et al. (44) reported successful clinical experience using modified tension band wire fixation to treat three cases of osteoporotic calcaneal avulsion fractures. All patients achieved bony union, with no re-displacement of fracture fragments during follow-up. This clinical study further verifies the effectiveness of tension band technology in treating calcaneal fractures. By converting the tensile force of the Achilles tendon into compression between fracture fragments, the tension band can effectively resist the risk of displacement. This study strongly confirmed the mechanical superiority of multi-screw configurations in resisting Achilles tendon traction, which is highly consistent with our finding that the Achilles tendon traction load in group C was effectively reduced through the use of the tension band technique.

Our study has several limitations, however. First, although CT data from a healthy adult were used and assigned osteoporotic bone material properties, this approach cannot fully replicate the characteristics of actual osteoporotic bone. In our study, the maximum load applied was 120 N (representing 10° dorsiflexion), yet during normal walking, the Achilles tendon bears up to three times one’s body weight, and during vigorous activity, it may reach 5,000 N. Thus, the conclusions of our study (e.g., displacement <0.1 mm, stress <100 MPa) apply only to the postoperative non-weight-bearing bed-rest phase and cannot be extrapolated to weight-bearing or rehabilitation periods. Second, as this study was based solely on finite element analysis, it cannot completely substitute for biomechanical testing; furthermore, the current model did not account for higher loading conditions or fatigue simulation. Third, the number of clinical cases was limited, and further randomized controlled trials are needed to more comprehensively evaluate the efficacy of different fixation constructs.

## 6 Conclusions

Combining clinical case analysis and finite element analysis, this study draws the following conclusions:

1. Among the five fixation constructs evaluated, the 180° annular plate demonstrated superior stress distribution and provided more stable fixation, effectively reducing compression of the fracture fragment on the overlying skin.
2. The use of the tension band technique helps convert the tensile force of the Achilles tendon into compressive force at the fracture site, which may reduce the risk of internal fixation failure.
3. The use of an appropriate surgical technique combined with rigorous postoperative management is fundamental to achieving successful clinical outcomes.

## Data Availability

The original contributions presented in the study are included in the article/Supplementary Material, further inquiries can be directed to the corresponding author.
